# HMGA1 silencing restores normal stem cell characteristics in colon cancer stem cells by increasing p53 levels

**DOI:** 10.18632/oncotarget.1914

**Published:** 2014-04-18

**Authors:** Francesca Puca, Marianna Colamaio, Antonella Federico, Marica Gemei, Nadia Tosti, André Uchimura Bastos, Luigi Del Vecchio, Salvatore Pece, Sabrina Battista, Alfredo Fusco

**Affiliations:** ^1^ Istituto di Endocrinologia ed Oncologia Sperimentale - CNR e/o Dipartimento di Medicina Molecolare e Biotecnologie Mediche, Università degli Studi di Napoli “Federico II”, Naples, Italy; ^2^ CEINGE, Biotecnologie Avanzate, Naples, Italy; ^3^ Istituto Europeo di Oncologia, Milan, Italy

**Keywords:** HMGA1, cancer stem cells, p53, colon carcinoma, NUMB

## Abstract

High-mobility group A1 (HMGA1) proteins are architectural chromatinic proteins, abundantly expressed during embryogenesis and in most cancer tissues, but expressed at low levels or absent in normal adult tissues. Several studies have demonstrated that HMGA1 proteins play a causal role in neoplastic cell transformation. The aim of this study was to investigate the role of these proteins in the control of cancer stem cells (CSCs), which have emerged as a preferred target in cancer therapy, because of their role in cancer recurrence. We observed that HMGA1 is overexpressed in colon tumour stem cell (CTSC) lines compared to normal and colon cancer tissues. We demonstrated that HMGA1 silencing in CTSCs increases stem cell quiescence and reduces self-renewal and sphere-forming efficiency (SFE). The latter, together with the upregulation and asymmetric distribution of NUMB, is indicative of the recovery of an asymmetric division pattern, typical of normal stem cells. We further found that HMGA1 transcriptionally regulates p53, which is known to control the balance between symmetric and asymmetric divisions in CSCs. Therefore, our data indicate a critical role for HMGA1 in regulating both self-renewal and the symmetric/asymmetric division ratio in CSCs, suggesting that blocking HMGA1 function may be an effective anti-cancer therapy.

## INTRODUCTION

Cancer arises from a small set of stem cells, or tumour-initiating cells, that differ from normal stem cells in their deregulated self-renewal and differentiation programs (1). Chemotherapy improves the 5-year survival of adult cancer patients by only 2.3% in Australia and 2.1% in the USA (2). Surrogate end point parameters such as ‘progression-free survival,’ ‘disease-free survival,’ or ‘recurrence-free survival’ reflect the temporary pause in the progression of the disease, seldom lasting more than a few months. Subsequently, the cancer typically returns with even more aggressive characteristics due to a few tumour-founding cells (the cancer stem cells or CSCs), which, because of their intrinsic chemoresistance, are spared and “naturally selected” by the routinely used anti-cancer drugs. This common trend makes the identification of CSC-specific targets and tightly related CSC-specific drugs necessary for the development of new effective anti-cancer therapies.

The High-Mobility Group A (HMGA) family includes three proteins: HMGA1a, HMGA1b, and HMGA2. These proteins are encoded by two distinct genes; the HMGA1a and HMGA1b proteins are products of the same gene through alternative splicing (3). The HMGA proteins bind the minor groove of AT-rich DNA sequences through their DNA binding domains, the so-called “AT-hooks.” HMGA proteins do not exhibit transcriptional activity *per se*, but they regulate the activity of several genes by interacting with the transcription machinery and altering the chromatin structure (4). The levels of HMGA proteins are low or absent in normal cells and adult tissues but are elevated in many tumours, neoplastically transformed cells, and embryonic cells (4). Their overexpression is largely associated with a highly malignant phenotype and also represents a poor prognostic marker, as HMGA overexpression often correlates with the presence of metastasis and reduced survival (5). Moreover, several studies indicate a causal role for *HMGA* gene expression in the process of carcinogenesis. Indeed, it has been reported that the blockage of their expression prevents thyroid cell transformation and promotes the death of malignant cells (6-7). Transgenic mice overexpressing either HMGA1 or HMGA2 develop uterine tumours, haematopoietic tumours, and pituitary adenomas (8-11).

The observation of HMGA1 upregulation in colon cancer dates back to 1996, when our group detected the HMGA1 proteins, previously called HMGI(Y), in human colorectal cancer cell lines and tissues but not in normal intestinal mucosa (12). Subsequently, we reported that HMGA1 protein expression was associated with the early stages of the neoplastic transformation of colon cells but only rarely with colon cell hyperproliferation (13), closely correlating with the degree of cellular atypia in adenomas. Very recently, Belton and colleagues (14) reported that HMGA1 overexpression induces cell proliferation and polyp formation in the intestines of HMGA1 transgenic mice and leads to metastatic progression and stem cell-like properties in colon cancer cells (14), suggesting that HMGA1 is a key regulator both in metastatic progression and in the maintenance of a stem cell-like state (14). Therefore, the aim of our study was to investigate the role of the HMGA proteins in colon cancer stem cells by silencing their expression.

Here, we report that HMGA1 silencing dramatically affects the survival of colon tumour stem cells and shifts stem cell division to an asymmetric pattern. The ability of HMGA1 to negatively regulate p53 promoter activity at the transcriptional level at least partially accounts for the effects induced by its inhibition on CTSCs.

## RESULTS

### HMGA1 is overexpressed in CTSCs and in the CD133+ sub-population

**Figure 1:**
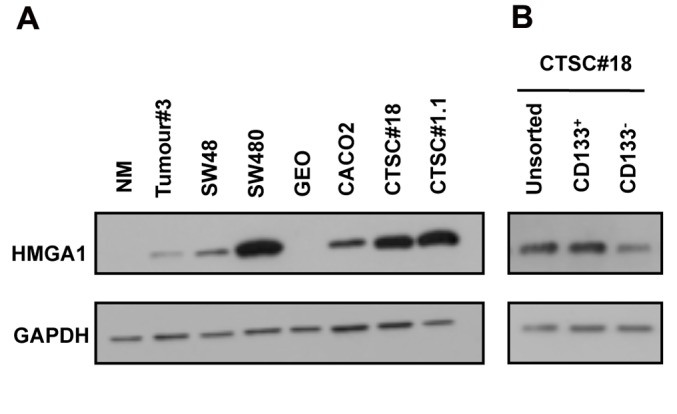
HMGA1 expression in CTSCs A) Western blot for HMGA1 in normal colonic mucosa (NM), colon cancer sample Tumour#3, colon tumour-derived cell lines (SW48, SW480, GEO, and CACO3), and colon tumour stem cells (CTSC#18 and CTSC#1.1). B) Western blot for HMGA1 in unsorted CTSC#18 and sorted CD133^+^ and CD133^−^ cells. GAPDH was used as a loading control.

We first analysed HMGA1 expression by western blot in normal colonic mucosa (NM), colon cancer, colon cancer cell lines and CTSC lines. As shown in Figure 1A, HMGA1 was undetectable in NM, whereas it was expressed in colon cancer (Tumour#3), in 3 colon cancer cell lines (SW48, SW480 and CACO2), and CTSCs (CTSC#18 and CTSC#1.1), which exhibited the highest HMGA1 expression. Interestingly, when CTSCs were stained for the cancer stem cell marker CD133 and then sorted, HMGA1 expression was enriched in CD133^+^ cells (Figure 1B). These data indicate that HMGA1 is overexpressed in CTSCs and is more abundant in stem cells than in precursors.

### HMGA1 knockdown impairs CTSC growth and induces apoptosis

**Figure 2:**
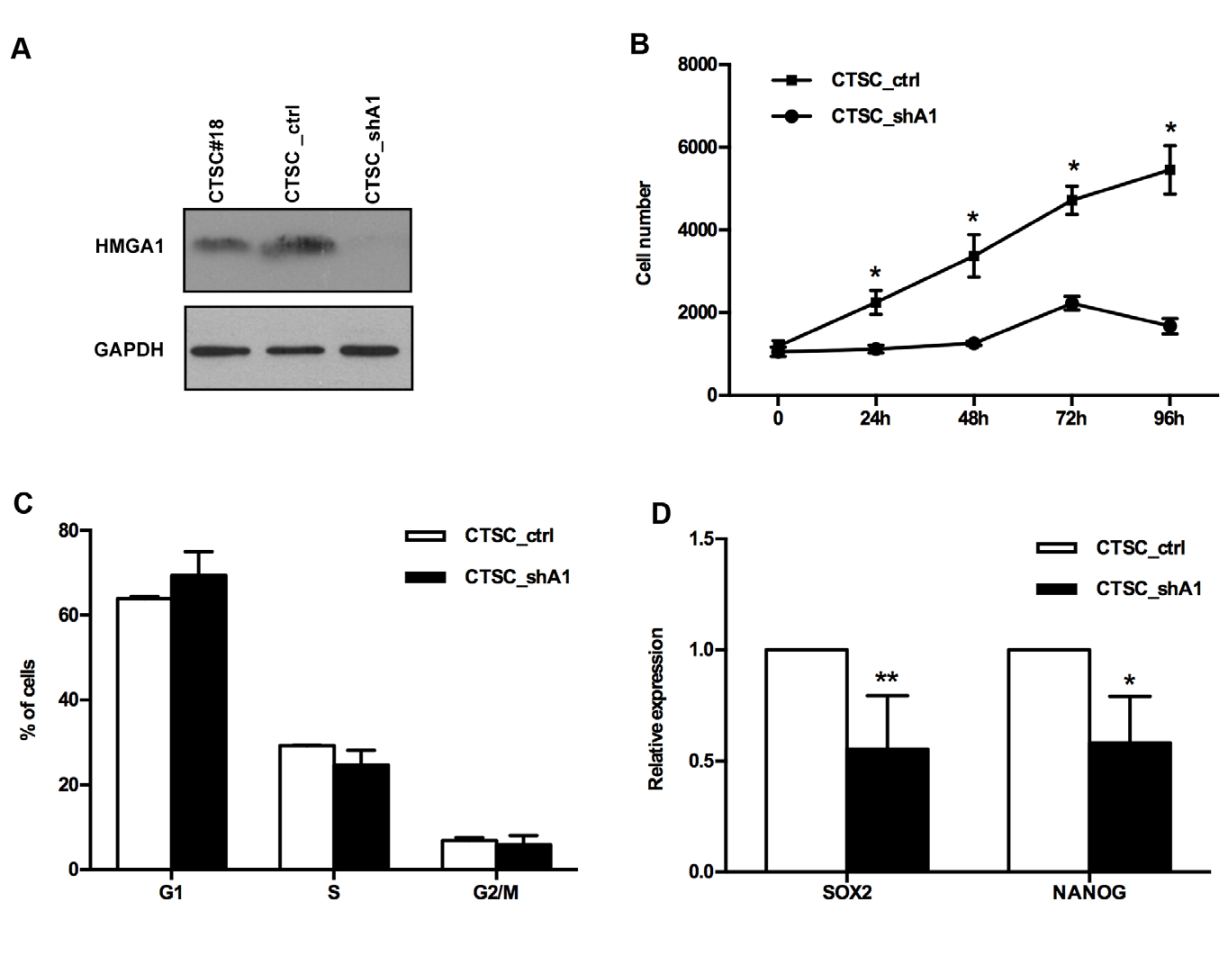
HMGA1 knockdown affects the proliferation and cell cycle of CTSCs A) Western blots for HMGA1 in untransfected, scramble-transfected (CTSC_ctrl) and HMGA1-knockdown (CTSC_shA1) cells. GAPDH is used as a loading control. B) Growth curve of stable scramble (CTSC_ctrl) and HMGA1-knockdown (CTSC_shA1) CTSCs. Data are the mean value ± SD of one representative experiment, performed in quadruplicate (*, p < 0.05, Mann-Whitney *U*-test). C) Histogram of the FACS analyses in CTSC_ctrl and CTSC_shA1 cells. Data are the mean value ± SD of 3 independent experiments. D) Relative expression of *SOX2* and *NANOG* gene expression in CTSC_ctrl and CTCS_shA1 cells, as determined by qRT-PCR. The expression level of each gene was normalized to the *G6PD* gene expression (*, p < 0.05; **, p < 1.01. Mann-Whitney *U*-test).

To understand the role of HMGA1 in CTSC, we silenced HMGA1 expression in the CTSC#18 cell line, using a short hairpin interfering construct (see the Materials and Methods section), leading to an HMGA1 knockdown efficiency of approximately 50%-80% in stable transfectants (Figure 2A). Growth curves performed on single-cell suspensions demonstrated that the knockdown of HMGA1 significantly reduced CTSC proliferation (p < 0.05) (Figure 2B). The analysis of cell cycle progression, performed by flow cytometric analysis, demonstrated that HMGA1 knockdown reproducibly altered cell cycle progression, inducing a mean increase of 5% in the G1 phase population and a concomitant mean reduction of 4% in the S phase (Figure 2C). As expected, HMGA1 knockdown reduced the expression of stem cell/pluripotency genes, such as SOX2 and NANOG (Figure 2D).

**Figure 3:**
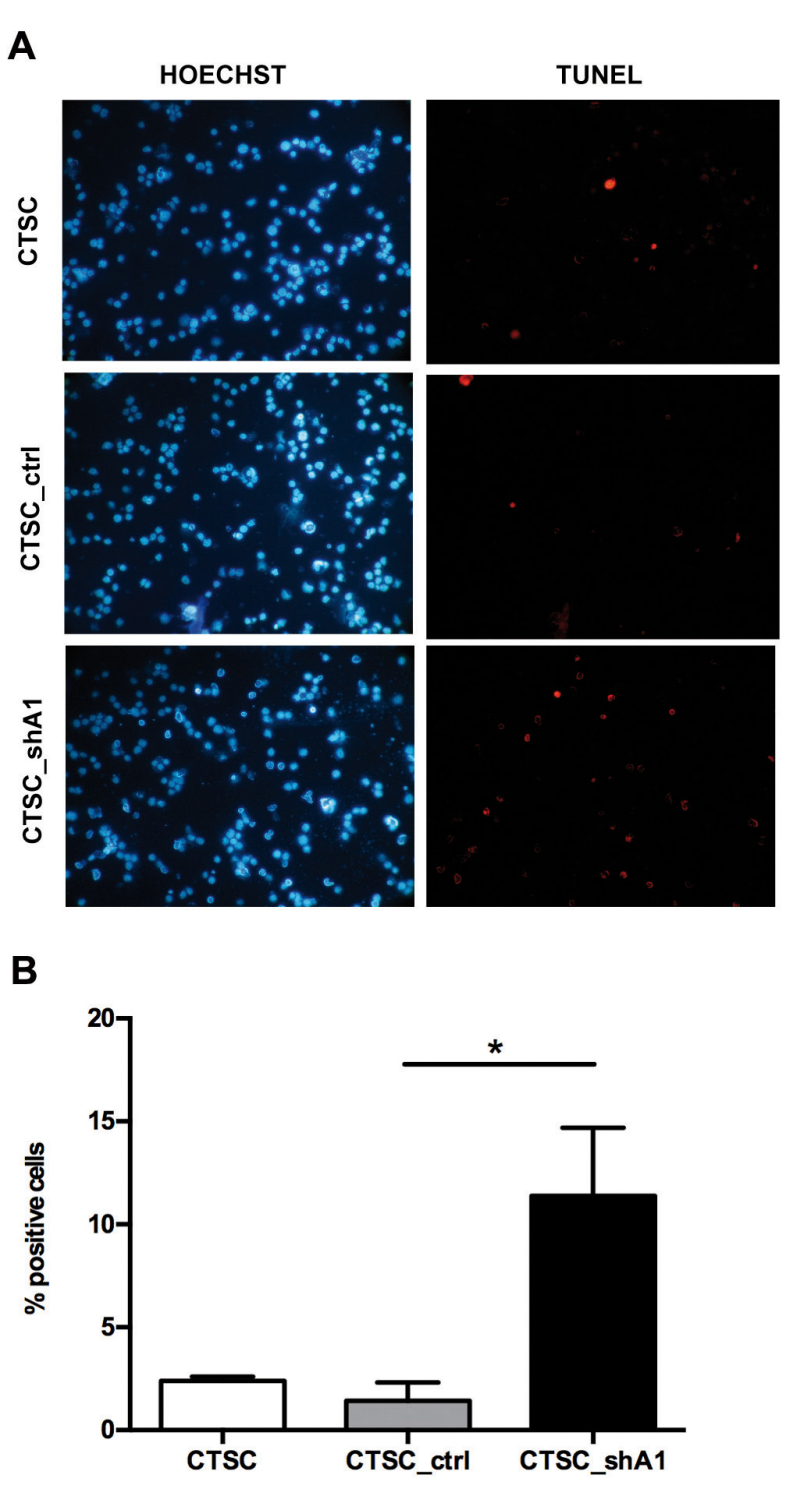
HMGA1 knockdown effects on apoptosis in CTSCs A) Fluorescence micrographs of TUNEL assays performed on non-transfected CTSCs (CTSC), CTSC_ctrl and CTSC_shA1 cells, double-stained with Hoechst dye (left) to identify total nuclei and with TMR red UTP (right) to identify apoptotic, TUNEL-positive cells. B) Bar chart representation of the number of TUNEL-positive cells per 100 Hoechst-positive nuclei in the samples shown in A. Each bar represents the mean ± SD of 10 arbitrary fields. An asterisk indicates the significance of the difference between CTSC_shA1 and CTSC_ctrl (*, p = 0.0014; Kruskal-Wallis test followed by Dunn's post-hoc test).

Because the alteration of the cell cycle only partially accounts for the reduction in CTSC proliferation induced by HMGA1 knockdown, we investigated apoptotic cell death. TUNEL (terminal deoxynucleotidyl transferase dUTP nick-end labeling) assays performed on stable transfectants exhibited a 7-fold increase in apoptotic cell numbers in CTSC_shA1 with respect to control cells (Figures 3A and B). These data suggest that, as in other cell systems (4), HMGA1 plays a key role in CTSC proliferation by affecting cell cycle progression and apoptosis.

### HMGA1 silencing impairs CTSC self-renewal and sphere-forming efficiency in serial passages

**Figure 4:**
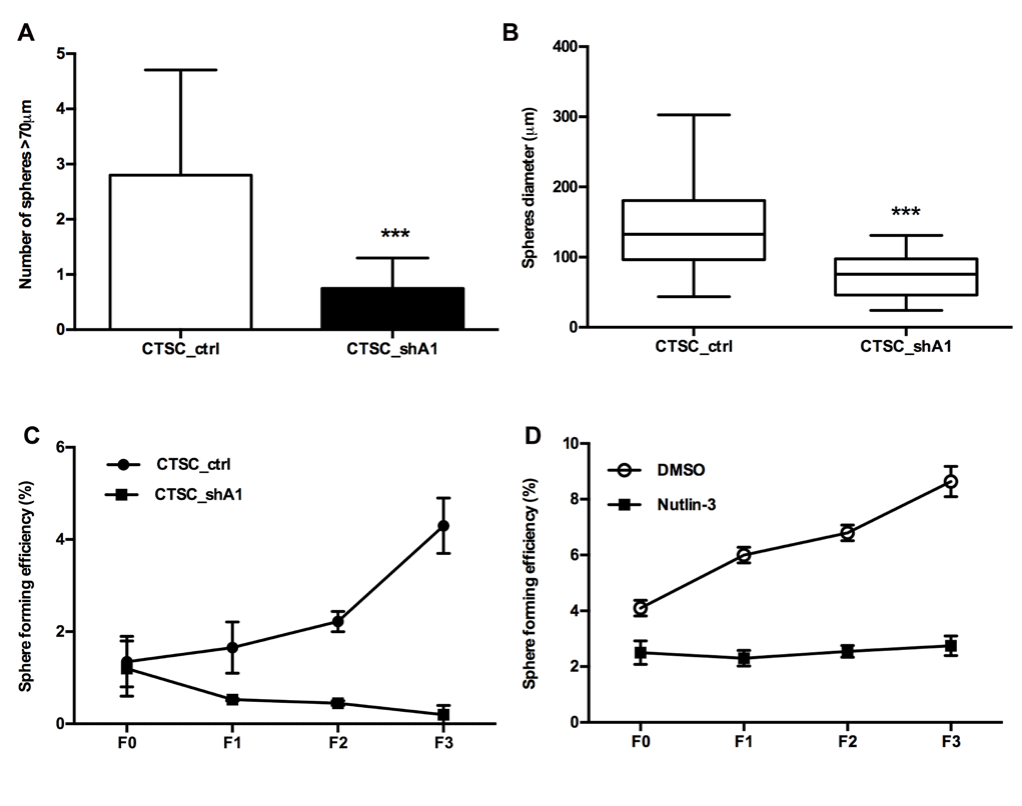
Effects of HMGA1 knockdown on the sphere-formation ability of CTSCs A) The diagram represents the average number of spheres in the methylcellulose-based medium after 7 days. Spheres with diameters > 70 µm were counted in each of 10 representative fields. Triple asterisks indicate the significance of the difference in the number of spheres formed by CSC_ctrl and CTSC_shA1 cells (***, p *<* 0.001; Mann-Whitney *U*-test). B) Diagram showing the sphere diameter distribution in CTSC_ctrl and CTSC_shA1 cells. Each bar represents the mean ± SD diameter found in 10 representative fields. (***, p < 0.001; Mann-Whitney *U*-test). C) Diagram showing the sphere-formation efficiency (SFE) ± SD in scramble- and HMGA1-knockdown CTSCs in serial passages (from F0 to F3). The spheres were disaggregated every 10 days. SFE is measured as the percentage of the number of spheres per plated cell at every passage. The data represent the results of two independent experiments. D) Sphere-formation efficiency (SFE) in parental CTSC#18 cells treated with DMSO or Nutlin-3 (5 µM) in serial passages (from F0 to F3). Spheres were disaggregated every 7 days. The data represent the mean value ± SD of two independent experiments.

CTSCs, as other types of cancer stem cells, are characterised by their ability to form spheres in suspension cultures (15) or in a semisolid medium. The number of spheres reflects the quantity of cells capable of *in vitro* self-renewal, whereas the number of cells per sphere measures the self-renewal capacity of each sphere-generating cell (16). Therefore, we assayed the ability of cells to form spheres in methylcellulose-based medium. A dramatic reduction in the number of spheres (Figure 4A) and in their diameter (Figure 4B) was observed in CTSC_shA1 cells compared with control CTSC_ctrl, thus indicating that HMGA1-interference affects CTSC self-renewal.

CSCs are known to differ from normal stem cells both in their deregulated self-renewal and cell division pattern, and an evaluation of their sphere-formation efficiency (SFE) in serial passages allows the assessment of the rate at which CSCs divide symmetrically (16-17). As shown in Figure 4C, the SFE of CTSC_shA1 cells decreased at every passage, whereas the number of spheres derived from parental (not shown) or CTSC_ctrl cells increased progressively. Interestingly, previous studies have demonstrated that the knock-out of p53 leads to increases in SFE and the symmetric division of mammary stem cells in serial passages, whereas the p53 stabiliser Nutlin-3 is able to reduce the SFE of ErbB2 mammary stem cells in serial passages (18). Consistent with these studies, we observed that the treatment of parental CTSC#18 with 5 µM Nutlin-3 was able to maintain the SFE constant over subsequent passages (Figure 4D), similar to what was observed for CTSC_shA1 cells. Conversely, SFE increased in DMSO-treated cells (Figure 4D). Therefore, these results indicate that HMGA1 silencing not only restores the ability to divide asymmetrically but also exhibits a more dramatic effect than p53 stabilisation alone.

### HMGA1 silencing induces quiescence and the asymmetric distribution of Numb in CSCs

**Figure 5:**
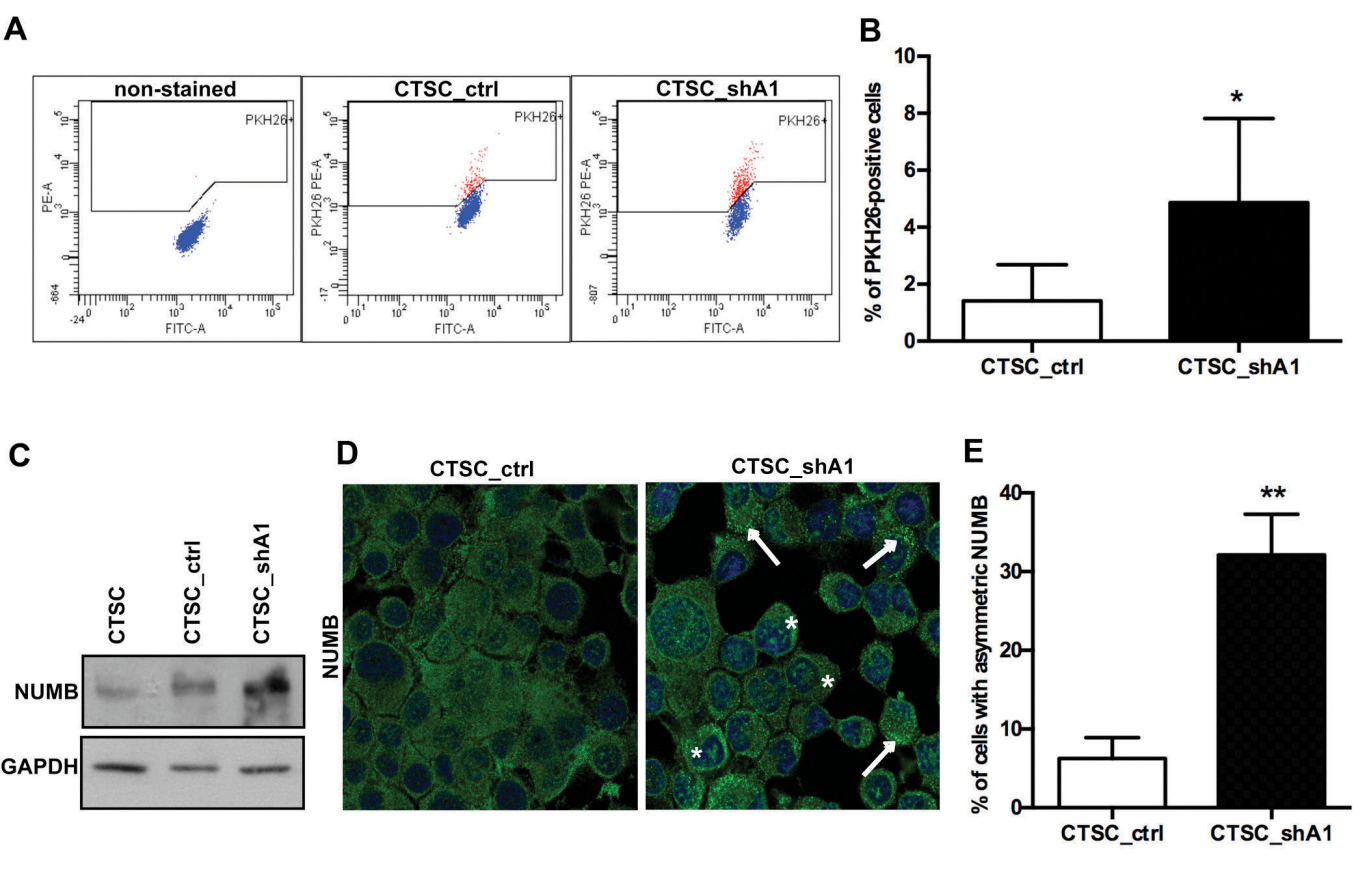
HMGA1 knockdown induces stem cell quiescence and asymmetric Numb distribution A) FACS plots of double-coloured (PKH26-phycoerythrin [PE] and fluorescein isothiocyanate [FITC-A]) CTSC_ctrl and CTSC_shA1 cells (one representative experiment). The left-most panel shows cells non-stained with PKH26, gated on physical parameters (forward scatter [FSC] and side scatter [SSC]) to exclude most of the debris and dead cells. B) Mean percentage of PKH26^bright^ cells in CTSC_ctrl and CTSC_shA1 populations, after 10 days from staining. Each bar represents the mean ± SD of 5 independent experiments (*, p < 0,05; Mann-Whitney *U*-test).C) Western blot for Numb in non-transfected, CTSC_ctrl and CTSC_shA1 cells. GAPDH was used as a loading control D) Immunofluorescence for Numb in CTSC_ctrl (left) and CTSC_shA1 (right) cells. The nuclei are stained in blue with DAPI. Arrows denote the crescent-shaped Numb distribution, whereas asterisks indicate nuclear-localised Numb. The histogram shows the percentage of cells with crescent-shaped, asymmetric Numb distribution, obtained in immunofluorescence analyses, as in C). Data are the mean value ± SD of 7 arbitrary fields for each sample. (** , p = 0.0048; Mann-Whitney *U*-test).

Long-term label retention of PKH26 dye is frequently used as an indicator of normal stem cell quiescence (19). Indeed, rapidly and symmetrically dividing CSCs tend to quickly lose PKH26 (18), which irreversibly binds to the lipid bilayers on cell membranes and is equally distributed among daughter cells during each cell division. Conversely, normal quiescent stem cells divide asymmetrically in one proliferating progenitor and one self-renewing PKH26-retaining quiescent stem cell. Therefore, we stained CTSC_shA1 and control CTSC_ctrl cells with PKH26 and performed FACS analyses after 10 days. As shown in Figure 5A and B, knockdown of HMGA1 expression led to a drastic increase in PKH26^bright^ cells (1.5 % in CTSC_ctrl cells versus 4.8 % in CTSC_shA1 cells), suggesting that the reduction in HMGA1 expression confers properties of quiescence to the stem cell compartment. Interestingly, very similar results were obtained when HMGA1-knockdown brain tumour stem cells (BTSCs) were stained with PKH26 (Colamaio et al., manuscript in preparation).

Asymmetric cell division can be demonstrated by the asymmetric partitioning of the cell fate determinant Numb (19). During asymmetric cell division, the part of the cell that inherits high levels of Numb will undergo differentiation, whereas the part that inherits low levels will produce a stem cell (20); conversely, in symmetrically dividing CSCs, Numb is expressed at lower levels and is uniformly distributed in such a way that both daughter cells will be equal in terms of Numb expression and function (20).

Therefore, we analysed Numb expression/distribution in HMGA1-knockdown and control cells. Western blot analysis for Numb revealed higher levels of Numb in CTSC_shA1 cells with respect to untransfected and CTSC_ctrl cells (Figure 5C). Moreover, immunofluorescence of the spheres (Figure 5D) demonstrated that whereas Numb was distributed almost uniformly in the cell cytoplasm of CTSC_ctrl cells, it formed an asymmetric punctuate crescent close to the cell membrane (Figure 5D, right panel, arrows) in CTSC_shA1 cells, where it also underwent partial nuclear localisation (asterisks). The frequency of Numb crescents increased from approximately 6% in control cells, to approximately 32% in CTSC_shA1 cells (Figure 5E). Together, these data demonstrate that HMGA1-silencing restores normal stem cell properties in CSCs, such as quiescence and asymmetric division.

### HMGA1 regulates p53 expression at the transcriptional level

**Figure 6:**
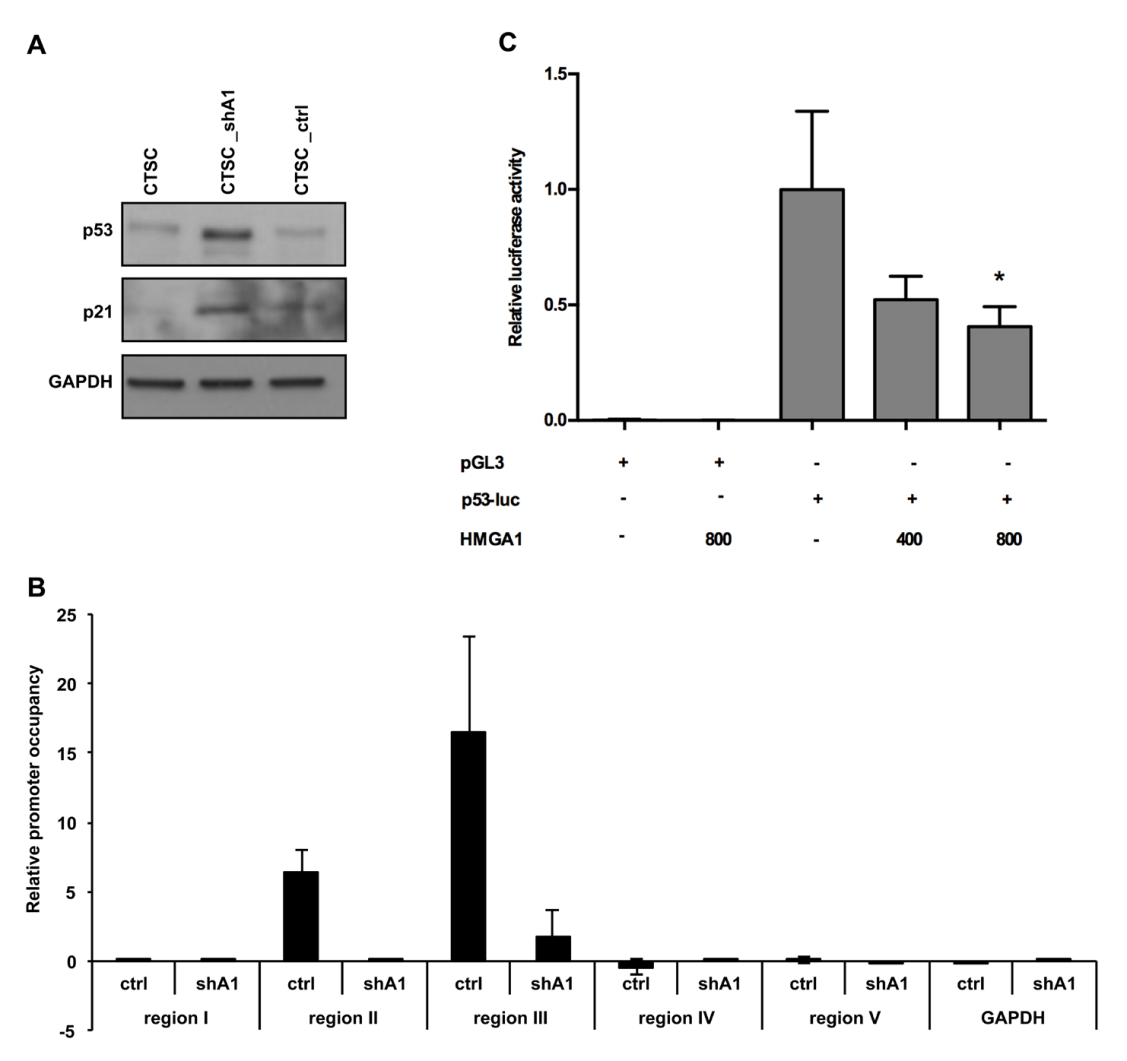
HMGA1 negatively regulates p53 expression at the transcriptional level A) Western blot analysis for p53 (upper panel) and p21 (middle panel) expression in non-transfected, HMGA1-knockdown, and scramble-transfected cells. GAPDH was used as a loading control. B) ChIP assay, detecting the *in vivo* binding of HMGA1 to the 5 sub-regions in the p53 promoter in CTSC_ctrl and CTSC_shA1 extracts. The relative occupancy of the p53 promoter regions by HMGA1 is indicated as vertical bars. GAPDH promoter amplicon was used as a negative control C) Luciferase activity of the p53 promoter in HEK293 cells in the presence or absence of an HMGA1-expressing vector. The amounts of the HMGA1-expressing vector are indicated. The data are the results of three independent experiments performed in duplicate. The relative luciferase activity was normalised with *Renilla* luciferase and was expressed as the fold induction over the activity of the p53 promoter (*, p < 0.05). pGL3-basic activity in the presence or absence of the HMGA1-expressing construct was used as a negative control.

Because it has been previously shown that an imbalance between asymmetric and symmetric division can be determined by loss of the p53 tumour suppressor (18), we analysed p53 expression in CTSC_shA1 cells. Western blots performed on untransfected, scramble-transfected, and HMGA1–knockdown CTSCs demonstrated that the downregulation of HMGA1 increased p53 protein expression (Figure 6A, upper panel) and was associated with an increase in the p53-regulated p21 protein (Figure 6A, middle panel), indicating the presence of a functional p53.

Subsequently, to assess the direct action of HMGA1 on p53 transcription, we evaluated HMGA1 protein binding to the *p53* promoter *in vivo* by performing ChIP assays. Therefore, CTSC_ctrl and CTSC_shA1 cells were cross-linked and immunoprecipitated with anti-HMGA1 or anti-IgG antibodies. Immunoprecipitation of chromatin was then analysed by quantitative PCR, using primers spanning 5 different regions of the p53 promoter (see the Materials and Methods section). Occupancy of the p53 promoter regions II and III by HMGA1 was clearly detectable (Figure 6B), whereas no amplification was observed in regions I, IV, and V, indicating the specificity of the binding to regions II and III (Figure 6B). Conversely, CTSC_shA1 cells exhibited a negligible enrichment for regions II and III (Figure 6B) and no enrichment at all for the remaining regions. These results indicate that HMGA1 protein binds the p53 promoter region *in vivo*.

Finally, to define the functional importance of HMGA1 binding to the p53 promoter, we evaluated the activity of the p53 promoter in the presence or absence of HMGA1, by performing luciferase assays. Therefore, HEK293 cells were transfected with a reporter construct, carrying the human p53 promoter (21) or a control backbone vector (pGL3-basic) in the presence of an HMGA1-expressing construct. As shown in Figure 6C, HMGA1 repressed the p53 promoter, and this effect was dose-dependent. Consistently, this repressive effect was abolished by the pharmacological blocking of the HMGA1-DNA interaction with distamycin (not shown), a drug known to displace HMGA1 from its AT-rich DNA regions (22). Therefore, these results clearly demonstrate that HMGA1 transcriptionally regulates p53 expression.

## DISCUSSION

CSCs are a distinct subset of cancer cells with the ability to self-renew through symmetric division and to generate a repertoire of various cell types, thus initiating and perpetuating tumour growth. CSCs are often endowed with distinctive surface markers, such as CD133, which, in colon cancer, in gliomas and other cancers, decorates tumour-initiating cells versus cells that are unable to initiate tumours in transplantation experiments (23). In human cancers, CSCs have been demonstrated to be a major cause of cancer treatment resistance, invasion, metastasis, and relapse. Thus, eliminating cancer cells with stem cell properties is of prime importance for the successful treatment of cancer, regardless of the tissue of origin.

Recent studies have suggested the critical role of the HMGA1 protein as a master regulator in both cancer stem cells (14; 24) and normal embryonic stem cells (25; 24). Indeed, HMGA1 has recently been shown to reprogram somatic cells into pluripotent stem cells by inducing stem cell transcriptional networks (26). Moreover, several studies have directly or indirectly demonstrated that HMGA proteins are tightly associated with stemness and play a critical role in the epithelial-mesenchymal transition (EMT) (27).

Here, we present data unveiling a central role for HMGA1 in CSC self-renewal. First, we demonstrated that HMGA1 is enriched in CTSCs compared with colon tumours and cancer cell lines and is more abundantly expressed in CD133^+^ cells than in CD133^−^ cells, strengthening its association with tumour-initiating cells.

Next, we demonstrated that the knockdown of HMGA1 by antisense methodology in colon CSCs induces a drastic reduction in cell proliferation due to a slight increase in the G1 phase population and a more evident increase in apoptosis, consistent with our previous findings (7). Moreover, CTSC_shA1 cells regain two properties typical of normal adult stem cells: an increased percentage (and brightness) of PKH26-positive cells, which indicates increased quiescence, and reduced SFE, suggestive of the recovery in asymmetric division at the expense of symmetric self-renewing division (17). This shift in the division modality, which is supported by the increase in the asymmetric distribution of NUMB, might be of practical clinical relevance because asymmetric division plays a tumour-suppressive role (20). Notably, we have achieved comparable results, in terms of SFE reduction, PKH26 increase and NUMB distribution in HMGA1-knockdown BTSCs (Colamaio et al., manuscript in preparation), suggesting the critical role of the HMGA1 protein in the regulation of CSCs of different tissue origin.

It has been demonstrated that ErbB2 transgenic mice harbour CSCs with an increased frequency of self-renewing divisions, higher replicative potential, and a preponderance of symmetric versus asymmetric division, highlighted by the symmetric distribution of the cell fate determinant Numb, compared with stem cells from wild-type mice (18). In these cells, Nutlin 3-mediated stabilisation of p53 was able to restore asymmetric division (as evidenced by the recovery of the asymmetric distribution of Numb) (18). The striking similarity of the phenotype obtained in CTSC_shA1 with Nutlin-3 treated CSCs and the tight association of HMGA1 and ErbB2 expression (28) prompted us to investigate the effect of HMGA1 interference on the p53-Numb axis. Indeed, we demonstrated that in CTSC_shA1 cells, the expression levels of both p53 and its downstream target p21cip are increased with respect to control cells. Moreover, we found that HMGA1 directly binds to a discrete region in the p53 promoter, and this binding has functional consequences, leading to p53 transcriptional downregulation.

It is worth noting that HMGA1 overexpression may lead to p53 inactivation by other mechanisms, such as through interfering with p53-mediated transcription of the p53 effectors BAX and p21 (29), transcriptionally activating the p53 inhibitor MDM2, and cytoplasmically relocalising its proapoptotic activator HIPK2 (30; 4). Therefore, the restoration of p53 function exerted by HMGA1 knockdown likely accounts for the effects of HMGA1 silencing on CSC cells.

However, we cannot exclude the possibility that in addition to a p53-mediated mechanism, HMGA1 silencing might affect CSCs through other mechanisms. In fact, we have demonstrated that whereas the SFE of Nutlin-3-treated CTSCs remains constant overtime, the SFE of HMGA1-knockdown cells progressively decreases in serial passages.

Since it has been shown that NUMB is able to interact with and stabilise p53 (31), the increased expression of NUMB observed in CTSC_shA1, concomitant with its nuclear relocalisation (F. Puca, unpublished preliminary results) would also enhance the stabilisation of p53. Therefore, taken together, these data suggest the existence of an HMGA1-Numb-p53 axis in which HMGA1 could play a significant role in regulating self-renewal either by directly acting on p53 and NUMB or by inhibiting their interaction in the nucleus. In conclusion, the results presented herein indicate that HMGA1 has a specific role in making decisions about stem cell fate, and thus, targeting HMGA1 may represent a promising CSC-specific anti-cancer strategy.

## MATERIALS AND METHODS

### Cell cultures and culture conditions

Colon tumour stem cell lines were kindly donated by Prof. Ruggero De Maria (Istituto Superiore di Sanità, Rome, Italy) and have been described elsewhere, together with their culturing conditions (15). Cytokines added to the medium included recombinant human FGF-basic and EGF (Peprotech, Rocky Hill, NJ). HEK293 cells were maintained in DMEM medium containing 10% fetal calf serum (Invitrogen).

### Colon samples

Normal and cancerous intestinal mucosa samples were kindly provided by Dott. Marina De Rosa (Facoltà di Medicina e Chirurgia, University “Federico II”, Naples, Italy) and have been described elsewhere (32).

### Quantitative reverse transcription PCR (qRT-PCR)

Total RNA was reverse transcribed using the QuantiTect® Reverse Transcription Kit (Qiagen), and qRT-PCR was performed by using Power SYBR® Green PCR Master Mix (Applied Biosystems, Foster City, CA, USA) according to manufacturer's instructions. *G6PD* was used to normalise RNA levels. The primers used were as follows: Hsa_HMGA1_Fw:5'-CAACTCCAGGAAGGAAACCA, Hsa_HMGA1_Rev: 5'-AGGACTCCTGCGAGATGC; Hsa_G6PD_Fw: 5'-ACAGAGTGAGCCCTTCTTCAA, and Hsa_G6PD_Rev: 5'-ATAGGAGTTGCGGGCAAAG; SOX2_Fw: 5'- GCACATGAACGGCTGGAGCAACG; SOX2_Rev: 5'-

TGCTGCGAGTAGGACATGCTGTAGG; NANOG_Fw: 5' - CAAAGGCAAACAACCCACTT; NANOG_Rev: 5'- TCTGGAACCAGGTCTTCACC. The 2^−ΔΔCt^ formula was used to calculate the differential gene expression.

### Immunostaining and cell sorting

For cell sorting, the cells were trypsinised, washed twice with PBS, and incubated with anti-human CD133 (CD133/2 (293C3)-PE, Miltenyi Biotech) for 20 minutes at 4°C. After washing twice with PBS, the cells were FACS sorted with a FACScan flow cytometer (Becton Dickinson, San Jose, CA) interfaced with a Hewlett-Packard computer (Palo Alto, CA).

### Western blots and antibodies

Total protein extraction, western blotting, and anti-HMGA1 antibodies have been described elsewhere (33).

The following other antibodies were used: anti-GAPDH (Santa Cruz Biotechnology, CA, USA) and anti-p21 (Cell Signaling Technology, Inc., Danvers, MA). Anti-Numb antibodies were obtained from Abcam (Cambridge, UK) and were used at 1:5000. Blots were visualised using western blotting detection reagents (GE Healthcare, Buckinghamshire, UK).

### Plasmids

The hairpin RNA interference plasmid for human HMGA1 (pLKO.1-HMGA1, TRCN0000018949) and the scramble control pLKO.1-Puro plasmid (SHC002) were obtained from Sigma-Aldrich. The sequence of the short hairpin RNA targeting the human HMGA1 gene was 5′–CCGGCAACTCCAGGAAGGAAACCAA CTCGAGTTGGTTTCCTTCCTGGAGTTGTTTTT–3′, (shHMGA1 targets coding region positions 446-466 of HMGA1 mRNA transcript variant 2).

The pGL3-luci vector containing the p53 promoter was kindly provided by Prof. David Reisman (Center for Colon Cancer Research Tissue Repository, University of South Carolina, Columbia) (21).

The pCDNA3.1-HMGA1 expression vector has been described previously (25).

### Transfections

CTSCs were electroporated using the Neon® Transfection System (Invitrogen). Cells were trypsinised with TrypLE™ Express (GIBCO) and counted; 1 × 10^6^ cells were subjected to the electric field (1400 V, 20 msec; 1 pulse). After 48 h, CTSCs transfected with the short hairpin-expressing constructs were selected with puromycin (2 µg/µl).

### Growth curves and TUNEL assay

Approximately 5 × 10^3^ stably transfected cells were plated in 96-well plates. Cells were counted in triplicate at daily intervals with a Burker hemocytometer chamber.

A TUNEL assay was performed using the In Situ Cell Death Detection kit (Roche) according to manufacturer's instructions.

### Flow cytometry

After trypsinisation, the cells were washed in PBS and fixed in 70% ethanol. Staining for DNA content was performed with 0.1% NP-40, 50 µg/ml propidium iodide, and 25 µg/ml ribonuclease A for 20 min. For each measurement, 10,000 events were analysed. We used a FACScanto II flow cytometer (Becton Dickinson, San Jose, CA). Cell cycle data were analysed with the ModFit LT 2.0 software (Verity Software) in a semiautomatic analysis procedure. Briefly, we manually selected the cell population in an FSC versus SSC dot plot and discarded debris, and then we gated single cells in a PI-height versus PI-area dot plot, excluding all doublets. The MODFIT algorithm was used to analyse our files, calculating the percentages of cells in each cell cycle phase.

### Sphere formation assays

Sphere-forming assays in methylcellulose-based medium were performed as previously described (34) with some modifications. Briefly, medium containing 0.8% methylcellulose was used instead of liquid medium, and other conditions were the same as in liquid medium. Three percent methylcellulose was purchased from R&D Systems (Minneapolis, USA), and a stock solution was made of 2% methylcellulose in DMEM/F12. A final concentration of 0.8% methylcellulose in DMEM/F12 was used for cell culture. Approximately 2 × 10^4^ cells from disaggregated CTSC spheres were resuspended in a semisolid medium and plated in 6-well plates. After 7 days, the spheres were microscopically visualised, and the diameters were measured.

Serial passage experiments were conducted as described previously (18) with some modifications. Briefly, 5,000 cells from disaggregated CTSC spheres were plated on 150-mm poly-HEMA-treated cell culture plates. After 10 days, the spheres were disaggregated and re-plated at the same density. The sphere-forming efficiency (SFE) at each passage was obtained by calculating the percentage of the number of spheres divided by the number of cells plated.

### PKH staining and flow cytometric analysis

PKH staining was performed as previously described (19).

CTSCs were trypsinised, filtered through a 40-μm cell strainer, resuspended in PBS (approximately 500,000 cells/ml), labelled with PKH26 (Sigma, 10^−7^ M, 5 min), washed twice, and plated.

For the flow cytometric analysis of PKH26-stained cells, CTSCs were trypsinised, filtered through a 40-μm cell strainer, and resuspended in PBS at a concentration of 1×10^6^/ml. The cells were subdivided into 5-ml polystyrene tubes (Falcon, Becton Dickinson). The BD FACSAria cytometer, equipped with four excitation laser lines (633 nm, 488 nm, 405 nm, and 375 nm) (Becton Dickinson) was used for FACS analysis, and the BD FacsDIVA software was used for data analysis.

PKH26 staining was evaluated by selecting the appropriate cell population according to the following gating strategy: (i) cells were first gated on physical parameters (forward scatter [FSC] and side scatter [SSC]) to exclude most of the debris and dead cells; (ii) doublets and aggregates were eliminated using the FSC-area vs. FSC-height pattern. We gated 10-15% of the brightest PKH26+ cells in a PKH26 versus empty channel dot plot.

### Immunofluorescence

Whole spheres were centrifuged and fixed with 4% paraformaldehyde, permeabilised, with 0.1% Triton X-100 and 3% BSA, and stained with anti-Numb (kindly donated by Prof. Salvatore Pece and previously described (18), followed by anti-mouse Alexa 488 (Jackson ImmunoResearch Europe Ltd., Suffolk, UK) antibodies. Confocal analysis was performed with a Leica TCS SP2 AOBS microscope.

### Luciferase assay

Transfections for luciferase assays were performed in HEK293 cells using the Lipofectamine 2000 method (Invitrogen S.r.l., S. Giuliano Milanese, MI, Italy). Approximately 2 × 10^5^ cells were transiently transfected with 200 ng of pGL3-luci vector containing the p53 promoter (kindly provided by Professor David Reisman, Center for Colon Cancer Research Tissue Repository, University of South Carolina, Columbia) and with the indicated amounts of the pCDNA3.1-HMGA1 expression vector or the corresponding empty vector together with 0.5 μg of *Renilla*. Various amounts of the pCDNA3.1 plasmid were co-transfected to keep the total DNA concentration constant. Transfection efficiencies were normalised using *Renilla* luciferase expression assayed with the dual luciferase system (Promega Italia, Milan, Italy). All transfection experiments were performed in duplicate.

### Chromatin immunoprecipitation

CHIP was performed as described previously (35). As a negative control, ChIP experiments were performed with isotype-matched preimmune IgG. The promoter occupancy was calculated with respect to the input as the percentage of anti-A1-immunoprecipitated DNA subtracted from the IgG-immunoprecipitated DNA. The P53 promoter regions assayed for HMGA1 binding refer to the nucleotide sequence previously published (21) and are indicated as follows: Region I: nt 401-557; region II: nt 538-650; region III: nt 631-751; region IV: nt 767-890; and region V: nt 989-1090.

The primers for each region were as follows:

Prom_hQ_tp53_1_F: 5'-CAGGCTTCAGACCTGTCTCC

Prom_hQ_tp53_1_R: 5'-GCTTTCAGTACATGGAAACGTAA

Prom_hQ_tp53_2_F: 5'-CGTTTCCATGTACTGAAAGCAA

Prom_hQ_tp53_2_R: 5'-CCCTAACGTTTTCTCCCAGA

Prom_hQ_tp53_3_F: 5'-TCTGGGAGAAAACGTTAGGG

Prom_hQ_tp53_3_R: 5'-AAGGGTGGAAGGAAGAAAGC

Prom_hQ_tp53_4_F: 5'-GCAGGATTCCTCCAAAATGA

Prom_hQ_tp53_4_R: 5'-GAGGGTGCAGAGTCAGGATT

Prom_hQ_tp53_5_F: 5'-GTTGATGGGATTGGGGTTTT

Prom_hQ_tp53_5_R: 5'-AGCTACCTGCTCCCTGGAC

Prom_GAPDH_1F: 5'-CCCAAAGTCCTCCTGTTTCA

Prom_GAPDH_R: 5'-GTCTTGAGGCCTGAGTACG

### Statistical analysis

Statistical analyses were performed using the Kruskal-Wallis test or the Mann-Whitney *U*-test. If the Kruskal-Wallis test was positive (p < 0.05), a pairwise comparison of subgroups was performed using Dunn's post-hoc test.
